# A Pleistocene freshwater ichthyofaunal assemblage from central Argentina: What kind of fishes lived in the Pampean lagoons before the extinction of the megafauna?

**DOI:** 10.1371/journal.pone.0235196

**Published:** 2020-07-08

**Authors:** Sergio Bogan, Federico L. Agnolin, Marcos Cenizo, Daniel Tassara, Adrián Giacchino

**Affiliations:** 1 Fundación de Historia Natural “Félix de Azara”—Centro de Ciencias Naturales, Ambientales y Antropológicas, Universidad Maimónides, Ciudad Autónoma de Buenos Aires, Argentina; 2 Laboratorio de Anatomía Comparada y Evolución de los Vertebrados, Museo Argentino de Ciencias Naturales “Bernardino Rivadavia”, Ciudad Autónoma de Buenos Aires, Argentina; 3 CONICET, Buenos Aires, Argentina; 4 Museo Municipal de Ciencias Naturales “Pachamama”, Santa Clara del Mar, Buenos Aires, Argentina; Monash University, AUSTRALIA

## Abstract

This study contributes to the knowledge of continental fishes recovered from sedimentary successions corresponding to the Bonaerean Stage/Age (late mid-Pleistocene) in the locality of Centinela del Mar, General Alvarado County, Buenos Aires province, Argentina. At this site we describe fossil fishes from a palaeolagoon, including *Corydoras* sp., *Pimelodella* sp., *Rhamdia* sp., *Oligosarcus* sp., small undetermined characids, *Jenynsia* sp. and *Odontesthes* sp. The recovered ichthyofaunal assemblage comprises at least seven taxa of Paranaean lineage. The taxonomic composition of the palaeoichthyofauna is quite comparable to that presently found in Bonaerean Watercourses of the Atlantic Drainage ecoregion. This suggests that local ichthyofaunal communities have remained relatively stable since the late mid-Pleistocene.

## Introduction

The extensive flatlands extending along east-central Argentina (locally known as Pampas) are interrupted by a few scattered hills of the Ventania and Tandilia Systems. A major feature of the Pampas is their capacity to accumulate surface water, resulting in a distinctive landscape composed of numerous shallow water ponds scattered throughout the area [1].

During the Pleistocene, the Pampas harboured a particular assemblage of giant mammals, mainly belonging to lineages with a very long history of isolation and endemism in South America [2, 3]. They weighed more than 1 ton and are commonly referred to as megafauna [4, 5]. In the late Pleistocene this megafauna reached a peak of about 40 species from South American lineages, including Tardigrada, Cingulata, Notoungulata, Litopterna, and Holarctic lineages such as Proboscideans [6, 7]. These megammamals coexisted with large mammals weighing more than 44 kg which comprised carnivores (felids, canids and ursids), and perissodactyl and artiodactyl ungulates of Holarctic origin [6, 7]. Holarctic immigrants arrived to South America during the “Great Biotic American Interchange” that began in the late Pliocene [7, 8].

The Pleistocene of the Pampas was also characterised by a succession of climatic cycles (closely linked to glacial cycles in the Andes), dominated by cold and dry glaciar periods that alternated with shorter, warmer interglacial periods [9, 10, 11]. These relatively rapid climate shifts affected the composition and distribution of terrestrial vertebrates [12]. By the end of the Pleistocene, all megammamals and almost all large mammal species became extinct during the global “Late Quaternary Extinction Event” [13], possibly due to strong changes in climate and vegetation coupled with a negative anthropogenic impact (e.g. [12, 14, 15, 16, 17, 18, 19, 20, 21]).

Most of the palaeontological studies conducted in the Pampas are based on mammalian fauna, and there is little information on vertebrates such as fishes and frogs, which are known to be sensitive to climate changes (see [22]). This contribution focuses on the fish assemblage recovered from the Centinela del Mar fossil site, located in the Pampas, southern Buenos Aires Province, Argentina. Our work represents a starting point to understand the effect of climatic changes on fossil fishes and their evolution in the Pampas during the Pleistocene.

The fossil record of continental fishes from the Pleistocene of Argentina is quite scarce and mainly restricted to outcrops of the Buenos Aires Province and, to a lesser extent, to the Santa Fe and Formosa Provinces [23, 24, 25, 26]. In the Pampean Region, fish remains have been described from seven fossiliferous localities: Cantera Vita, Centinela del Mar, Arroyo Azul, Paso Otero, Río Quequén Salado, Río Reconquista, Cantera Los Sauces and Salto [23, 27, 28, 29, 30, 31].

The fossiliferous locality of Centinela del Mar has yielded a variety of late Middle Pleistocene ichthyofauna, including Perciformes [32], Atheriniformes [33], Characiformes [34] and Cyprinodontiformes [35].

The aim of the present contribution is to report new fossil fish records for Centinela del Mar, placing them in a regional context. It is important to emphasize that we provide the first description of anatomical elements belonging to Siluriformes and to previously reported groups in the studied area. In addition, we include a list of all fossil freshwater fishes recovered from this locality and discuss the implications of our findings for the evolution of fish assemblages in the Pampean Region.

Based on the available taxonomic and sedimentary information on the fossil-bearing units, we compare the recovered taphocenosis and the modern biocenosis in the area. Our analysis attempts to provide a first step in understanding the genesis and palaeoecology of the palaeolagoon that would have been inhabited by these fishes, and to propose new tools for a historical interpretation of the present-day distribution pattern of fishes in the Plain between Hills area of the Pampean Region.

## Materials and methods

The fossil fishes described here were collected in fieldtrips included in the Project “Análisis bioestratigráfico y paleoambiental de los afloramientos fosilíferos de la localidad de Centinela del Mar, provincia de Buenos Aires, Argentina (late Pliocene-middle Pleistocene)”, National University of La Plata, Buenos Aires Province, Argentina (Ref. 1000-03215-2005). Remains were recovered under strict stratigraphic control using detailed longitudinal and vertical profiles [36]. The relatively large remains were collected by direct observation using a non-selective procedure, while subfacies with a high concentration of small specimens were detected applying prospecting techniques on microareas throughout the entire outcrop. The sediment was sieved through a series of four stacked sieves of downwardly decreasing mesh size (2000, 1000, 500 and 250 μm), which were immersed in water to increase the dissolution of the cementing material in a uniform way. Finally, specimens were recovered in the laboratory by hand-picking under a binocular microscope (Leica Ms5).

Taxonomic identification was complemented by detailed comparisons with osteological collections of extant specimens housed in the ichthyological collection at Fundación de Historia Natural “Félix de Azara” (CFA-IC).

### Institutional abbreviations

**CFA-IC**, Ichthyological Collection, Fundación de Historia Natural ‘Félix de Azara,’ Buenos Aires, Argentina; **MLP**, Vertebrate Palaeontological Collection, Division Paleontología de Vertebrados, Museo de La Plata, La Plata, Argentina; **MPH-P**, Palaeontological Collection, Museo de Ciencias Naturales de Miramar, Miramar, Argentina.

### Geological settings

**Fig 1:**
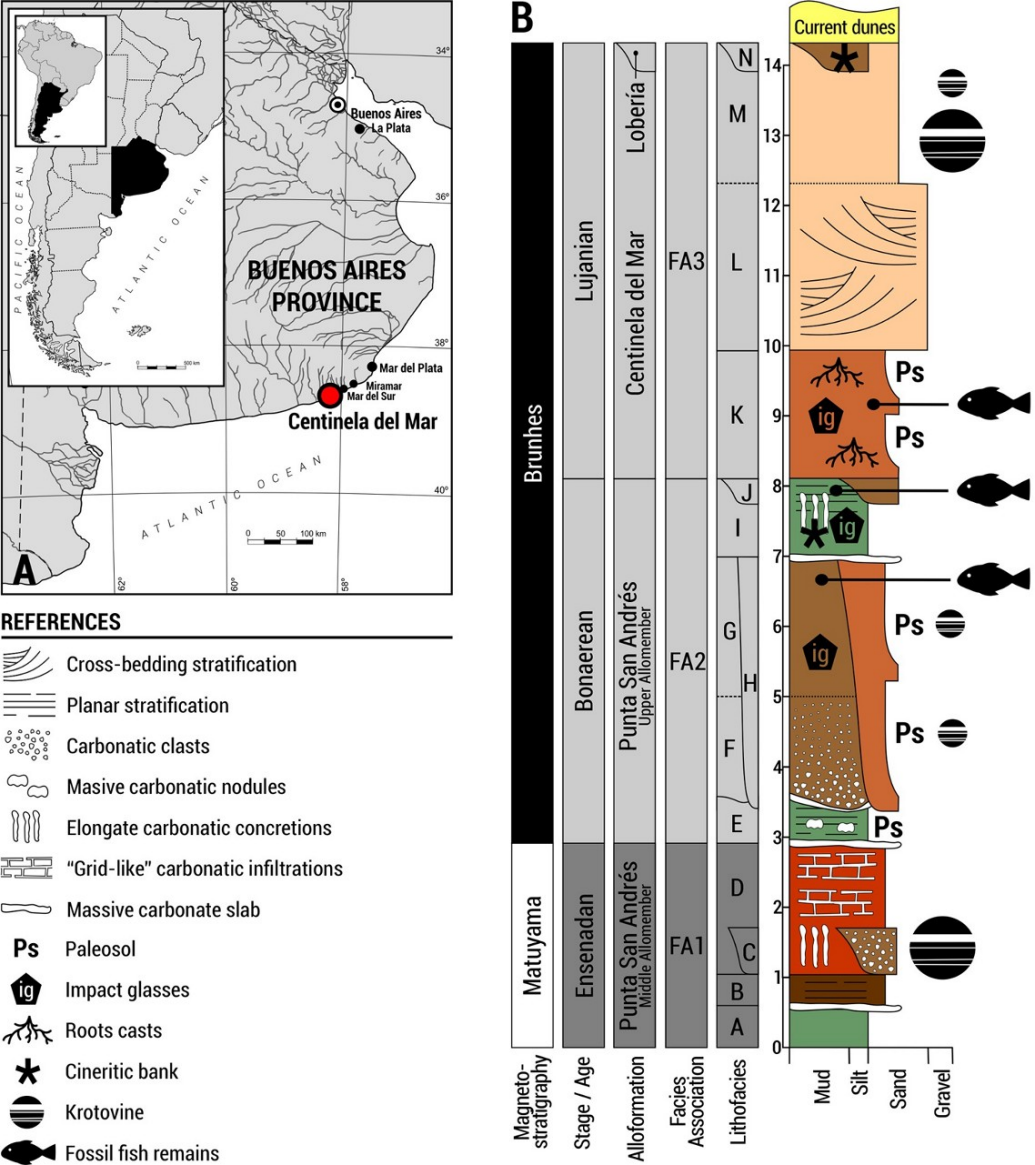
A. Geographic location and B, integrated stratigraphic section of Centinela del Mar locality (General Alvarado County, Buenos Aires Province, Argentina).

Centinela del Mar (38° 26´ S, 58° 14´ W) is located at the SE coast of Buenos Aires Province, in the marine littoral of the General Alvarado County (Fig 1). In this locality there is a coastal cliff along the shoreline ranging from 5 to 15 m in height and extending for about 2.85 km; it is bounded to the SW by the confluence of El Pescado, Nutria Mansa and Malacara streams.

The sedimentary succession exposed in the area was described and analysed in detail by Cenizo [36], who established a stratigraphic section composed of 14 lithofacies grouped into four facies associations that correspond to three alloformations.

**Fig 2:**
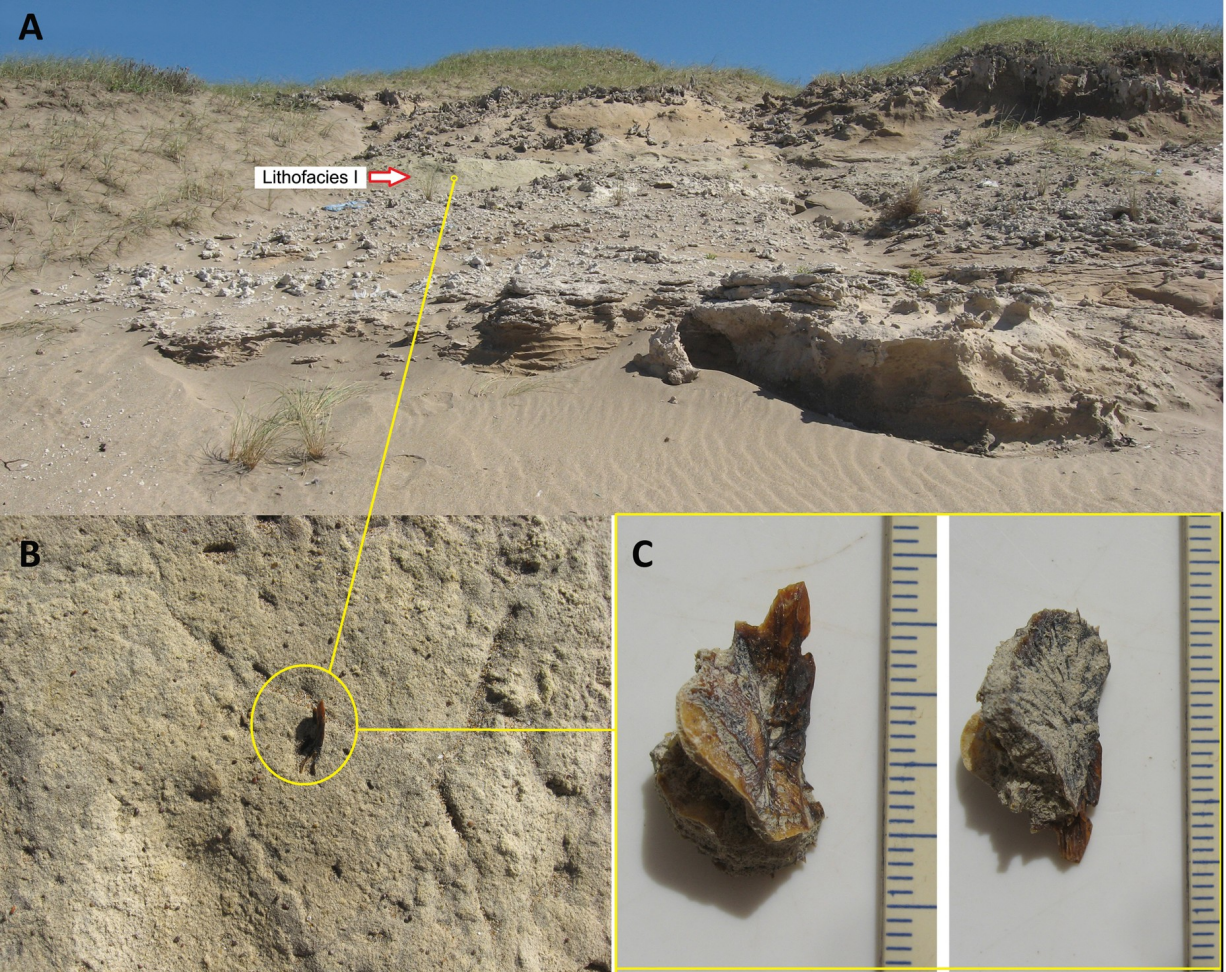
A, Coastal cliff showing detail of Lithofacies I (38° 25´ 30´´ S, 58° 10´ 48´´ W; B, *in situ* fish bones belonging to *Rhamdia* sp.; C, the same specimen of *Rhamdia* sp., cleaned.

The fish remains found in Centinela del Mar come from the Lithofacies G, I and K (Figs 1 and 2; hereafter Lf G, Lf I and Lf K [32,33,34,35], which were referred to as units E, G and I, respectively, in [37, 38]. These stratigraphically contiguous units are located in the upper-mid section of the local section and correspond to a prograding system of fluvio-lacustrine to estuarine-marine environments [31, 36]. The Lf I, located between Lf G and K, yielded the most important ichthyofaunal assemblage in terms of abundance and diversity, and this contribution is mainly focused on interpreting the fish assemblage recovered from this unit.

Both Lf G and Lf I are grouped into the Facies Association 2 (FA2, [36]). It was tentatively correlated with the unit Upper Allomember of the Punta San Andrés Alloformation [36], located about 50 km NE of the Centinela del Mar [39, 40]. These units are well exposed and extend all over the cliff surface of Centinela del Mar to the NE and SW [36, 40].

The Lf G is represented by 2 m of light brown sandy loessoid silts, generally with cross-bedding stratification. This body has mantle-like sedimentary geometry, showing a transitional basal contact with the underlying Lf F. The latter is represented by flow deposits with abundant carbonate clasts, extending throughout the regional outcrop [36]. The Lf G has meteorite impact-derived glasses (locally known as “*escorias*”), radiometrically dated (40Ar/39Ar) at 230±30 ka [41].

The overlying Lf I is a tabular deposit composed of 0.5–1.5 m of green to grayish-green sandy loessoid silts, whose roof is usually crowned by nodular and/or elongate carbonate concretions (Fig 2). Based on sedimentological and faunal proxies, its genesis might be linked to flood plains with development of lentic water bodies [36].

Finally, the Lf K is the basal unit of the Facies Association 3 (FA3), corresponding to the Alloformation Centinela del Mar [36]. It comprises 1–1.5 m of massive and friable reddish-brown sandy loessoid silts with some small fragments of crushed shells. The deposit shows a widespread tabular geometry and is separated from the underlying Lf I by an erosive discordance. The unit is affected by two palaeosols with preserved carbonate root casts and formicid nests [42, 36]. Sedimentary and faunal evidence indicate a genesis linked to a brackish environment, such as a salt marsh, coastal lagoon or an estuary [36, 43, 41], with the subsequent development of soils. Lf K also shows a concentration of impact glasses radiometrically dated (40Ar/39Ar) at 445±21 ka [41].

Cenizo [36] estimated possible chronological limits for the fossil-bearing units containing the analysed ichthyofauna based on geochronological data and stratigraphic correlations. In this way, the impact glasses found in Lf G and Lf K were dated at 230±30 ka and 445±21 ka, respectively [41]. This reflects a stratigraphic incongruence between the ages obtained, as upper glasses appear to be older than lower ones [36]. Hence, although these results should be interpreted with caution, maximum ages for these deposits can be tentatively determined.

On the other hand, specimens of *Tegula patagonica* dated by U/Th at 93.5 ± 3.5 ka were recovered from beach facies exposed in the cliffs of the Faro de Claromecó locality (Tres Arroyos County; [43]). Taking into account this information, Isla *et al*. [43] allocated these deposits to the “Belgranense” marine transgression (namely Pascua Formation) and attributed them to the Sangamonian interglacial (*ca* 130 ka, MIS 5e). These authors also assigned the facies of the littoral mixing zone of the FA 3 (Alloformation Centinela del Mar *sensu* [36]) present in Centinela del Mar, to the “Belgranense” transgression. The FA 3 includes the Lf K, which is the upper unit with fossil fish remains.

Following this criterion, the set of Lf G, Lf I and Lf K containing fossil ichthyofauna would span the geochronologic interval between *ca*. 230 and 130 ka, corresponding to the latest Bonaerean Stage (late Middle Pleistocene) and very close to the Bonaerean-Lujanian transition [29, 44, 45]. However, these dates should be viewed with caution until more accurate estimations become available.

### Regional hydrogeography

Centinela del Mar locality is within the Chaco-Pampean plain, in a peripheral central-eastern sector of the Bonaerean Positive morphostructural unit [46]. The latter comprises the geological provinces of Tandilia and Ventania, as well as the Bonaerean Plains between Hills area [47]. This plain, which includes the foothill areas of Ventania and Tandilia, slightly exceeds 200 m in the central sector between both mountain ranges and becomes lower towards the Atlantic Ocean [48, 49]. There are sinuous water courses showing narrow alluvial floodplains with variable flow regime (occasionally dependent on rainfall intensity) and permanent wider water courses flowing down from the hills. The Quequén Grande River is the main watercourse on this plain, and its valley is possibly under structural control [49], as it lies between the Tandilia and Ventania systems [50]. Both mountain systems are composed of Precambrian-Palaeozoic rocks (*e*.*g*., [46, 51]), and may have exhibited positive topography since the Middle Miocene [46], whereas the Plain between Hills area is covered by loess and limestone deposits dating from the Miocene to the Holocene. The absence of marine outcrops in the area suggests that the Bonaerean Positive behaved like an island during the Paranean marine transgression in the mid-Miocene [46, 52].

The tectonic peculiarities of the Plain between Hills area have led to a particular hydrogeographic pattern in the Chaco-Pampean system, which is known as Direct Tributaries of the Atlantic Ocean (in Spanish: *“Tributarios Directos del Océano Atlántico*”; [53]). These watercourses are entirely autochthonous, run sub-parallel to each other and perpendicular to the coastline, and drain into the ocean [53, 54]. Many of them have formed shallow lakes due to damming by coastal dunes [53, 55, 56]. Moreover, these watercourses have numerous waterfalls, cascades and ravines generally composed of poorly consolidated rocks [53, 36]. All these features characterise the watercourses of the Plain between Hills area as immature and developed under arid or semiarid climates [53].

Although the regional hydrogeographic pattern was shaped by the interaction of tectonic, climatic and eustatic factors, at least since the Miocene, the main features of the current configuration seem to date back to the late Pleistocene (i.e., Lujanian Stage; [51, 57, 58]. Therefore, this pattern may correspond to the post-Bonaerean uplift (*sensu* [59]), which began *ca* 40–30 ka [60] as the result of neotectonic activity [29, 51, 61, 49] and/or changes in sea level [62]. These phenomena remain to be fully elucidated [49].

### Ichthyofauna of the Bonaerean Plains between Hills area

The hydrogeographic characteristics of the Pampean hills have a deep influence on fish composition. In the Bonaerean Plain between Hills area of the Pampean Region, there are eastern coastal basins of a series of small and isolated rivers that drain directly into the Atlantic Ocean, as evidenced by the large overlap between the Direct Tributaries of the Atlantic Ocean area [53] and the icthyogeographic area known as Bonaerean Watercourses of Atlantic Drainage (in spanish *“Cursos Bonaerenses de Pendiente Atlántica”*; [63, 64]. Both areas are located at the southern end of the Brazilian Ichthyogeographic Subregion. These basins represent one of the southernmost distribution limits of Brazilian taxa in South America [65]. Fish communities belong to Paranaean lineages [65, 64] and show a low species diversity. Indeed, although the Brazilian Subregion supports the greatest ichthyofaunal richness in the world (with over 552 species in Argentinian basins) [63, 64, 65, 66], fish diversity decreases markedly towards the south of the Río de la Plata estuary. A total of 180 Brazilian species have been reported for Buenos Aires Province [67], 138 of which were found in the Paraná-Río de La Plata basin [67, 68]. Fish diversity decreases westwards (including tributaries) from 100 to 60 species and only a few of them occur in headwaters of tributaries [68, 69]. Likewise, about 33 species have been recorded southwards along the Salado River [64, 65]. This gradient of decreasing fish diversity reaches a minimum of 14 species at 200 km to the south of the Salado River [54, 64, 70, 71], within the icthyogeographic area of Bonaerean Watercourses of Atlantic Drainage. It has been proposed that the presence of some characids and poeciliids in the area may be the result of recent dispersal mediated by humans [54, 71, 72]. Moreover, *Synbranchus marmoratus* was only observed in headwaters of the Malacara stream [71], *Austrolebias monstruosus* in Nutria Mansa stream, *Cyphocharax voga* in Sauce Grande basin [73] and *Australoheros facetum* in a few basins [54]. In the Bonaerean Watercourses of the Atlantic Drainage, local fish communities are most often composed of two to four taxa, whereas a few larger water bodies hold up to six species [54]. The low richness of the Brazilian species in this area may not only be explained by current climate conditions, but also by the evolution of the topography and hydrographic net, which probably functioned as geographic barriers to species dispersal.

Although the watercourses of this drainage net are isolated from each other, they share almost the same ichthyofauna, suggesting that they were interconnected during the Pleistocene, when sea level was relatively low. At that time, the basins of the ichtyogeographic area under consideration were longer than nowadays and extended deep into areas that became submerged beneath the Atlantic Ocean [49, 54, 71, 73, 74, 75].

## Results

### Systematic paleontology

**Fig 3:**
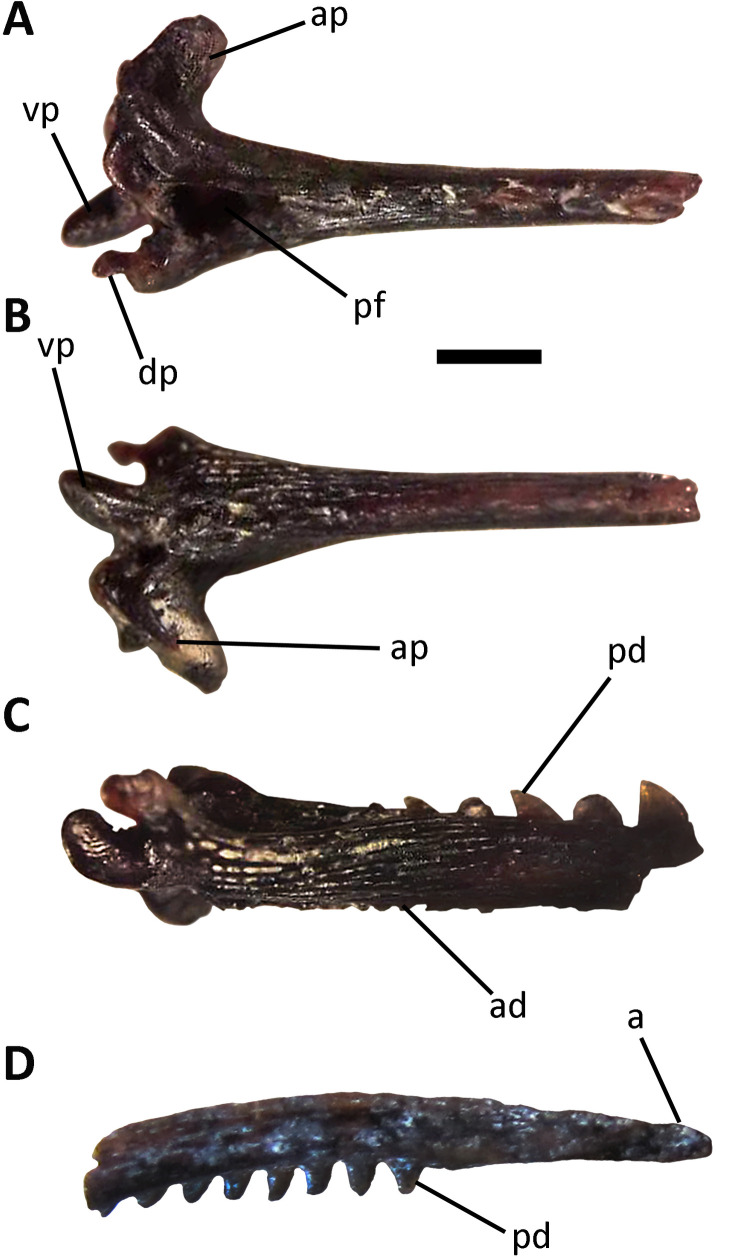
*Pimelodella* sp. A-C, right proximal pectoral spine (MPH-P 917); D, distal end of pectoral spine (MLP 04-V-2-502). A, posterior, B, anterior, and C, ventral, and D, dorsal views. **Abbreviations**: a, apex; ad, anterior denticles; ap, anterior process; dp, dorsal process; pd, posterior denticles; pf, posterior fossa; vp, ventral process. Scale bar: 1 mm.

Infraclass Teleostei Müller, 1845
Order Siluriformes Grande, 1987
Family Heptapteridae Gill, 1861
Genus *Pimelodella* Eigenmann & Eigenmann, 1888
*Pimelodella* sp.
(Fig 3A–3D)

#### Referred specimens

Proximal fragment of right pectoral spine and distal fragment of pectoral spine (MLP 04-V-2-502; Fig 3D); proximal fragment of right pectoral spine (MLP 04-V-2-503); and proximal fragment of right pectoral spine (MPH-P 917; Fig 3A–3C).

#### Occurrence

Facies Association 2, Lithofacies I (late Bonaerean Stage, late middle Pleistocene; [36]).

#### Description and comparisons

MLP 04-V-2-502, MLP 04-V-2-503, and MPH-P 917 correspond to proximal and distal fragments of right pectoral spines, constituted by the articular head, neck and part of the body. These are well-ossified elements, of sub-rectangular cross-section and compressed transversely. The dorsal and ventral surfaces of the spines exhibit parallel grooves and small pits. The anterior and posterior surfaces are dentate, with anterior denticles being numerous, small, straight, and exclusively restricted to the basal third of the spine. More than 37 anterior denticles are preserved in MLP 04-V-2-502 and about 10 denticles in MLP 04-V-2-503. The anterior denticles become progressively smaller towards the spine apex until they disappear, so that the anterior edge of the spine is completely edentate at its apical portion. At the proximal end, the anterior denticles tend to bend towards the ventral surface. The posterior surface of the spine bears posterior denticles directed towards the base of the spine. These are larger than the anterior denticles and occur on the dorsal surface of the spine.

The pectoral spines described herein show marked differences between the anterior and posterior denticles. The denticles on the anterior edge are small; they are regularly arranged and perpendicular to the surface at the base of the spine, while they bend at its proximal end. The posterior margin of the spine bears proportionally larger denticles that are straight or slightly directed towards the base of the spine. This combination of characters is typical of the genus *Pimelodella*, and has not been described for other Siluriformes [76, 77, 78]. The fossil spines described herein were compared with those of many specimens belonging to the extant southernmost distributed species of the genus (housed in CFA-IC), *Pimelodella gracilis* (n = 11) and *Pimelodella laticeps* (n = 22). The fossil specimens bear a close resemblance to *P*. *laticeps*, while they differ from *P*. *gracilis*. Like in *P*. *laticeps*, the anterior edge of the fossil spines at the level of the neck shows a marked irregularity in the dentate arrangement, a sinuous denticle row and many denticles tending to curve ventrally. However, the fragmentary state of the fossil spines and the existence of over 70 extant species of *Pimelodella* warn us to be cautious when identifying species.

The southern distribution limit of *P*. *gracilis* is located in the Río de la Plata River and tributary streams in Magdalena County, in the northeast of Buenos Aires Province [67, 68, 79], while that of *P*. *laticeps* is further south, in basins close to Bahía Blanca City, in the southwest of Buenos Aires Province [54, 68, 79].

In Argentina, the fossil record of specimens assigned to the genus *Pimelodella* is restricted to the late Pleistocene of the Buenos Aires Province [27, 28, 80, 81].

**Fig 4:**
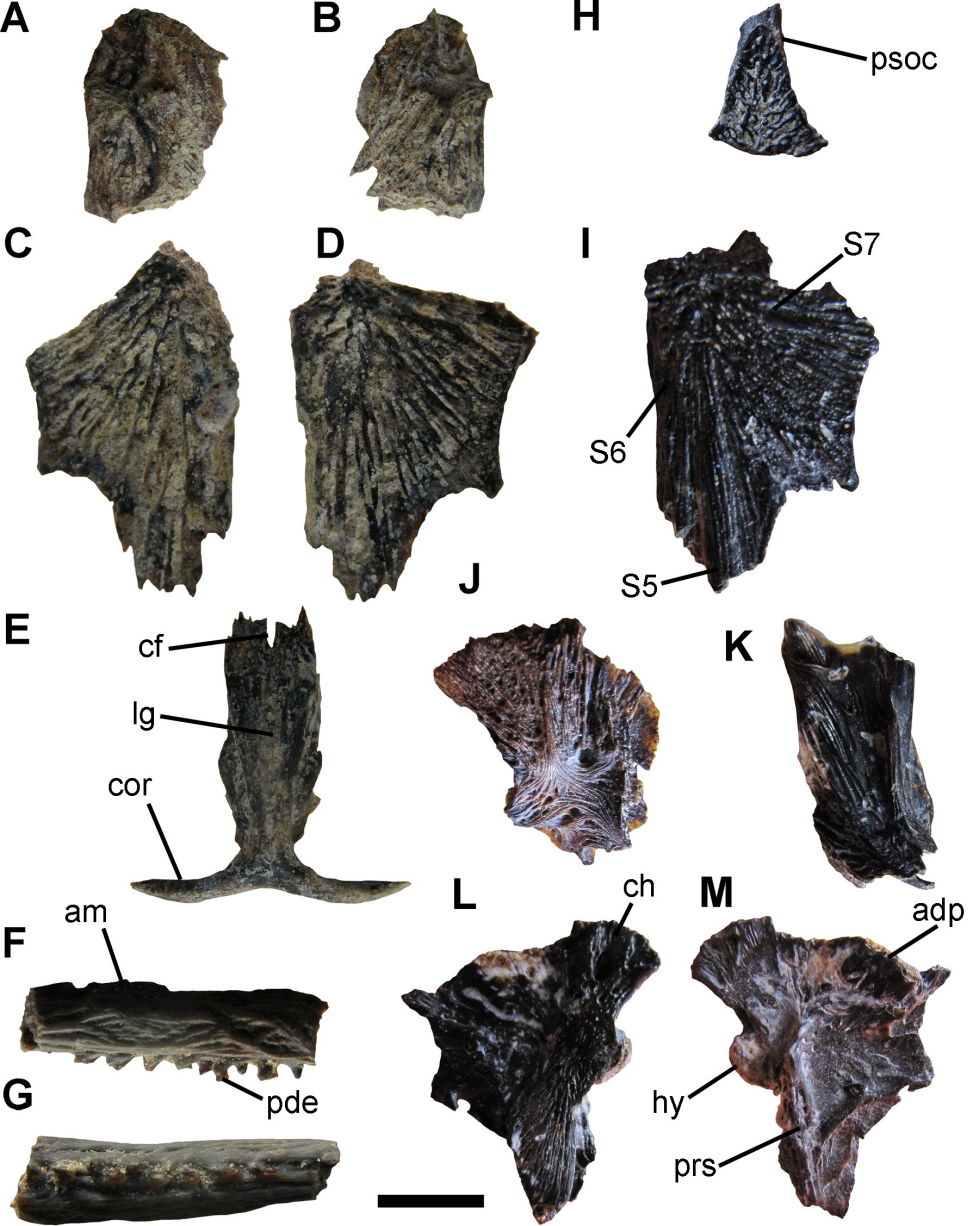
*Rhamdia* sp. A-E, specimen MPH-P 918; F-G, specimen MPH-P 919; H-M, specimen MLP 04-V-2-386. A, right pterotic in dorsal view; B left pterotic in dorsal view; C, right frontal in dorsal view; D, left frontal in dorsal view; E, mesethmoid in dorsal view; F,G fragment of pectoral spine in F, side and G, posterior views; H, fragment of parietosupraoccipital in dorsal view; I, left frontal in dorsal view; J, lateral ethmoid in dorsal view; K, right fragment of cleithrum in lateral view; L,M, right hyomandibular in L medial, and M, lateral views. Scale bar: 10 mm. **Abbreviations**. adp, anterodorsal process; am, anterior margin; cf, anterior cranial fontanelle; ch, chondral articulation cor, cornua; pde, posterior denticles; hy, hyomandibular condyle for articulation with the opercle; lg, longitudinal groove; prs, scar for articulation with the preopercular; psoc, posterior process of the parieto-supraoccipital; S5-7, pores 5, 6 and 7 of the epiphyseal branch of the supraorbital sensory canal.

Genus *Rhamdia* Bleeker, 1858
*Rhamdia* sp.
(Fig 4A–4M)

#### Referred specimens

Proximal fragment of left pectoral spine (MLP 04-V-2-504); proximal fragment of left pectoral spine (MLP 04-V-2-505); fragment of pectoral spine (MPH-P 919; Fig 4F–4G); anterior portion of left frontal right lateral ethmoid, posterior process of the supraoccipital, fragment of right opercle, complete hyomandibular, fragment of right cleithrum, articular portion of the coracoid, urohyal (MLP 04-V-2-386, associated elements from the same individual; Fig 4H–4M); mesethmoid, both frontals and both pterotics from the same individual (MPH-P 918, associated elements from the same individual; Fig 4A–4E).

#### Occurrence

Facies Association 2, Lithofacies I (late Bonaerean Stage, late middle Pleistocene; [36].

#### Description and comparisons

The pectoral spines MLP 04-V-2-504, MLP 04-V-2-505, and MPH-P 919 are well ossified, strong, and of subcircular cross-section. The dorsal and ventral surfaces of both specimens show many subparallel grooves and small pits, with irregularly interwined striations and rugosities generating an anastomosed ossification pattern. The anterior edge is edentate in its proximal third and distally it has denticles perpendicular to the longest axis of the spine. The posterior margin bears denticles that are straight to somewhat directed to the apex of the spine. These are irregular and occasionally bicuspid.

MLP 04-V-2-386 includes a set of associated though unarticulated ossified elements, belonging to a large individual. The preserved urohyal has a subtriangular outline and shows a distinctive longitudinal keel in dorsal view and two conspicuous articulation facets for the hypohyals at the anterior end. This element exhibits a conspicuous central foramen in ventral view and near its anterior end. The surface of the fragment of the cleithrum shows a dense ornamentation of thin bony striations; this element is robust, and bears a marked concavity for articulation with the pectoral spine in ventral view. The outer surface of the frontal is ornamented with numerous and deep striations radiating from the centre. In dorsal view, the frontal has the pores 5, 6 and 7 (S5, S6 and S7, respectively) of the epiphyseal branch of the supraorbital sensory canal. The S5 is placed at the anterior end, the S6 is close to the medial edge, whereas S7 is close to the central axis of the frontal (Fig 4). The lateral ethmoid, has a rectangular outline showing a posterior area densely ornamented with well-defined striations in dorsal view. The parieto-supraoccipital is represented by a fragment of the posterior process showing a subtriangular outline with the anterior base getting progressively narrower towards the distal end. Like most of the skull roof elements, there is a well-developed ornamentation of striations on the dorsal surface. The chondral articulation extends two-thirds its length on the dorsal margin of the hyomandibular. A wide scar for the articulation with the preopercular extends along the posteroventral surface of the bone, reaching the condyle for articulation with the opercle. The antero-dorsal process of the hyomandibular is well developed, transversely laminar and acute, and bears a foramen for the facial nerve at its antero-ventral margin.

MPH-P 918 includes associated elements from the same individual, most of which correspond to the anterior region of the skull already described above. The mesethmoid is elongate and laterally compressed in the region of contact with the nasal bones; it shows a shallow but well-defined longitudinal groove and has long and slender cornua. The mesethmoid is deeply concave at the anterior margin, while it shows an irregular edge corresponding to the suture line with the frontal at the posterior margin. In its middle line, it has a deep notch representing the anterior margin of the anterior cranial fontanelle.

Within the Heptapteridae, the described pectoral spines (MLP 04-V-2-504, MLP 04-V-2-505, and MPH-P 919) are morphologically similar to those of species belonging to the genus *Rhamdia*, sharing the following traits: (1) advanced ossification, proximal end of subrectangular cross-section becoming progressively sub-circular towards the distal end; (2) outer surface very irregular and run by numerous grooves with small pits opening at their bottom; (3) anterior margin of the fourth proximal edentate; and (4) posterior dentate with single row of irregular denticles,occasionally with more than one tip per base [76, 78].

On the other hand, the skull elements (MLP 04-V-2-386, MPH-P 918) could be assigned to the genus *Rhamdia* based on the following combinations of characters: (1) outer ornamentation composed of deep striations radiating from centre of frontal (this condition is shared with species of the genus *Brachyrhamdia* [31, 78, 82, 83]); (2) parieto-supraoccipital robust, with subparallel bony ridges running on its outer surface, radiating from the base of the occipital process; (3) supraorbital sensory canal of frontal with four ramifications ending in pores opening to the exterior; (4) opening for the epiphyseal branch of the supraorbital sensory canal (S6) relatively small; and (5) frontal orbital margin short and slightly concave [31, 82, 83].

In Argentina, the fossil record of *Rhamdia* is restricted to the late Pleistocene of Buenos Aires Province [24, 27, 28, 31, 81]. Accordingly, specimens assigned to this genus have been reported from sediments referable to the late Pleistocene (*ca*. 20–22 ka BP; [84], in the Gruta do Ioiô cave, Bahía State, northeastern Brazil.

**Fig 5:**
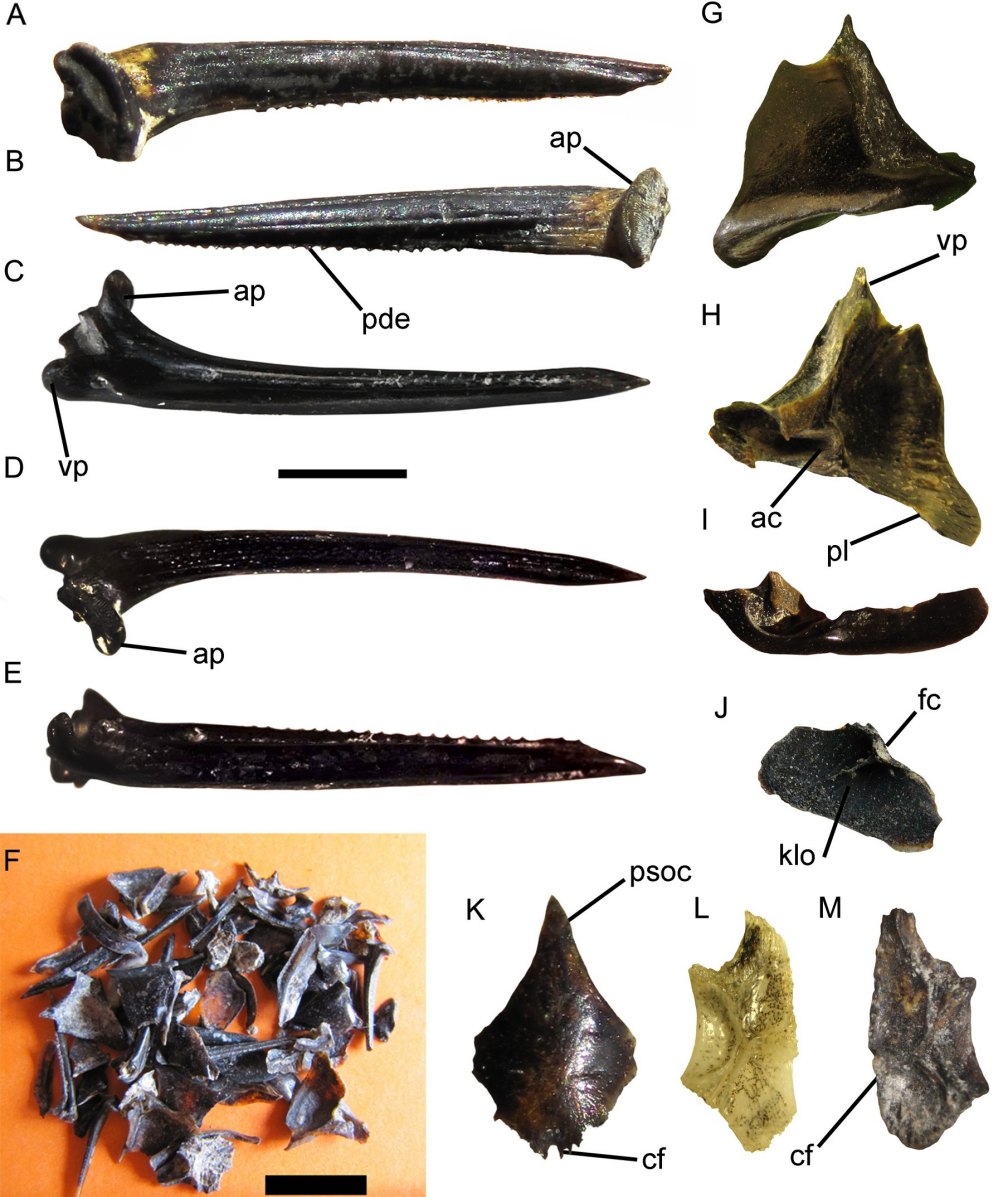
*Corydoras* sp. A-E, pectoral spines, A (MLP 04-V-2-455), B (MLP 04-V-2-488), C-E (MPH-P 915) in A,B,D, anterior; C, posterior; and E, lateral views; F, isolated fossil bones found after sieving of the sediment; G-I, cleithrum (MLP 04-V-2-364); J, opercle (MPH-P 916); K, parieto-supraoccipital bone (MLP 04-V-2-471) in dorsal view; L-M frontals L, (CFA-IC-9218) comparative extant *Corydoras paleatus* and M, (MLP 04-V-2-360) *Corydoras* sp. Scale bar: 5 mm (A-E, G-M) and 10 mm (F). **Abbreviations**: ac: articular cavity; ap, anterior process; cf, cranial fontanelle; dp, dorsal process; fa: facet for articulation; klo: keel for insertion of levator operculi; pde, posterior denticles; psoc, posterior process of parieto-supraoccipital; pl: posterior lamina laterally; vp, ventral process.

Family Callichthyidae Bonaparte, 1838
Genus *Corydoras*, Lacépède, 1803
*Corydoras* sp.
(Fig 5A–5M)

#### Referred specimens

Almost all skeletal elements of several individuals of *Corydoras* were recovered: frontals (MLP 04-V-2-48, MLP 04-V-2-360 to 362, MLP 04-V-2-486; Fig 5K and 5M); parieto-supraoccipitals (MLP 04-V-2-345 to 348, MLP 04-V-2-471, Fig 5G, MLP 04-V-2-383, MLP 04-V-2-499 to 501); postemporal-supracleithrum (MLP 04-V-2-509 to 511); pectoral spines (MLP 04-V-2-318 to 344, MLP 04-V-2-372 to 382, MLP 04-V-2-436 to 438, MLP 04-V-2-455, MLP 04-V-2-460 to 462, MLP 04-V-2-466 to 469, MLP 04-V-2-488, MLP 04-V-2-491 to 495; MPH-P 915; Fig 5A–5E); coracoids (MLP 04-V-2-459, MLP 04-V-2-463 to 465, MLP 04-V-2-490, MLP 04-V-2-497); cleithra (MLP 04-V-2-352 to 359, MLP 04-V-2-363, MLP 04-V-2-364, MLP 04-V-2-368 to 371, MLP 04-V-2-431 to 435, MLP 04-V-2-472 to 485); dorsal spines (MLP 04-V-2-365 to 367, MLP 04-V-2-489, MLP 04-V-2-496); small pre-adipose spine (MLP 04-V-2-506); lateral plates (MLP 04-V-2-384); nuchal plate (MLP 04-V-2-507, MLP 04-V-2-508) and opercles (MLP 04-V-2-349 to 351, MLP 04-V-2-470, MLP 04-V-2-498; MPH-P 916; Fig 5J).

#### Occurrence

Facies Association 2, Lithofacies G and I (late Bonaerean Stage, late middle Pleistocene; [36]).

#### Description and comparisons

*Corydoras* is the most abundant of the taxa recovered from the locality of Centinela del Mar. A large number of specimens are represented by all the skeletal elements and therefore only some elements of taxonomic importance are described herein.

The parieto-supraoccipital is laminar and slightly convex dorsally. It has a well-developed posterior process of spine-like morphology and subtriangular outline. At the anterior end it shows an irregular edge corresponding to the suture line with the posterior margin of the frontals. An open notch representing the posterior margin of the anterior cranial fontanelle is present in its middle line.

The frontal is relatively flat, with smooth surface and of sub-rectangular outline. It has a small opening of the cranial fontanelle at the post-medial margin. The supraorbital canal runs longitudinally inside the frontal and opens through the epiphyseal pore located centrally. The orbital margin is strongly concave.

The opercle is dorsoventrally high and rounded in outline, with a strongly angulated posterior margin. The bone bears a facet for articulation with the condyle of the hyomandibular at the anterodorsal end. This is also observed in the family Callichthyidae, whereas it occupies a more dorsal position in Loricariidae and Astroblepidae [85]. On the inner surface, a bony keel for insertion of the *m*. *levator operculi* is directed ventrally, which is considered a diagnostic character for the Corydoradinae [86]; such keel is directed dorsally in the remaining members of Callichthyidae [86, 87]. On the outer lateral surface, the bone has numerous bases for odontoid insertion, a plesiomorphic condition in Corydoradinae [87].

The morphology of the cleithrum resembles that of other Corydoradinae, and is unique among the Loricarioidei. It has a well-developed and anteriorly expanded vertical lamina, with an important vertical process in its dorsolateral region [86].

The pectoral spines have a sharp-pointed apex. The spine body is slightly curved and shows posterior denticles that are almost perpendicularly oriented to the main axis of the spine and extend along the entire margin. The anterior surface is edentate but it generally shows numerous ring-like structures for the insertion of odontoid. The dorsal surface of the spine shows bases for the insertion of odontoid scattered on the body of the spine. The dorsal spines are slightly curved, large and strongly ossified, with the apex directed backwards. The posterior body of the spine shows a conspicuous groove containing small teeth, which runs through the spine from the apex to the base. The centrum of the articular head is pierced by a hole in anteroposterior direction.

A large number of lateral bony plates covering the body surface have been preserved. These plates are laminar, slender, elongate and of subrectangular outline. On their anterior surface there is a longitudinal facet where adjacent scales overlap.

The remains described herein can be assigned to *Corydoras* based on numerous shared characters. The recovered bony plates are indistinguishable from those covering the body of extant Corydoradinae species by being subrectangular and markedly elongate. The recovered pectoral spines are extremely acute, a distinctive feature of the subfamily Corydoradinae, in contrast to the robust pectoral spines with blunt apex characterising the sufamily Callichthyinae [85]. Moreover, these materials most probably belong to the Corydoradinae by having small pores without elevated rings for insertion of odontoid.

On the other hand, the presence of thin frontal bones in cross-section is a synapomorphic condition for Corydoradinae [86, 87]. The frontal fontanelle of the genus *Corydoras* is longitudinally elongated, while that of the genus *Aspidoras* is markedly reduced [86, 87]. The supraoccipitals lack the posterior cranial fontanelle as in *Corydoras* [88], while it is present in *Aspidoras* [86, 87].

The recovered material is clearly referable to the subfamily Corydoradinae, showing strong affinities with the extant species of the genus *Corydoras*, to which the material is referred.

Among the species of the genus, the spines are very similar to the extant species *Corydoras paleatu*s in having perpendicularly directed serrations along the entire posterior margin of the pectoral spines (vs. serrations directed towards pectoral spine base, serrations directed towards pectoral spine tip, perpendicularly directed serrations, if present, bifid or restricted to proximal region of pectoral spine [89]). However, the isolated nature of the available elements precludes any unequivocal specific identification.

Remains referable to the subamily Corydoradinae are very abundant in the Quaternary outcrops of Argentina, particularly in the Pampean Region [24, 27, 28].

**Fig 6:**
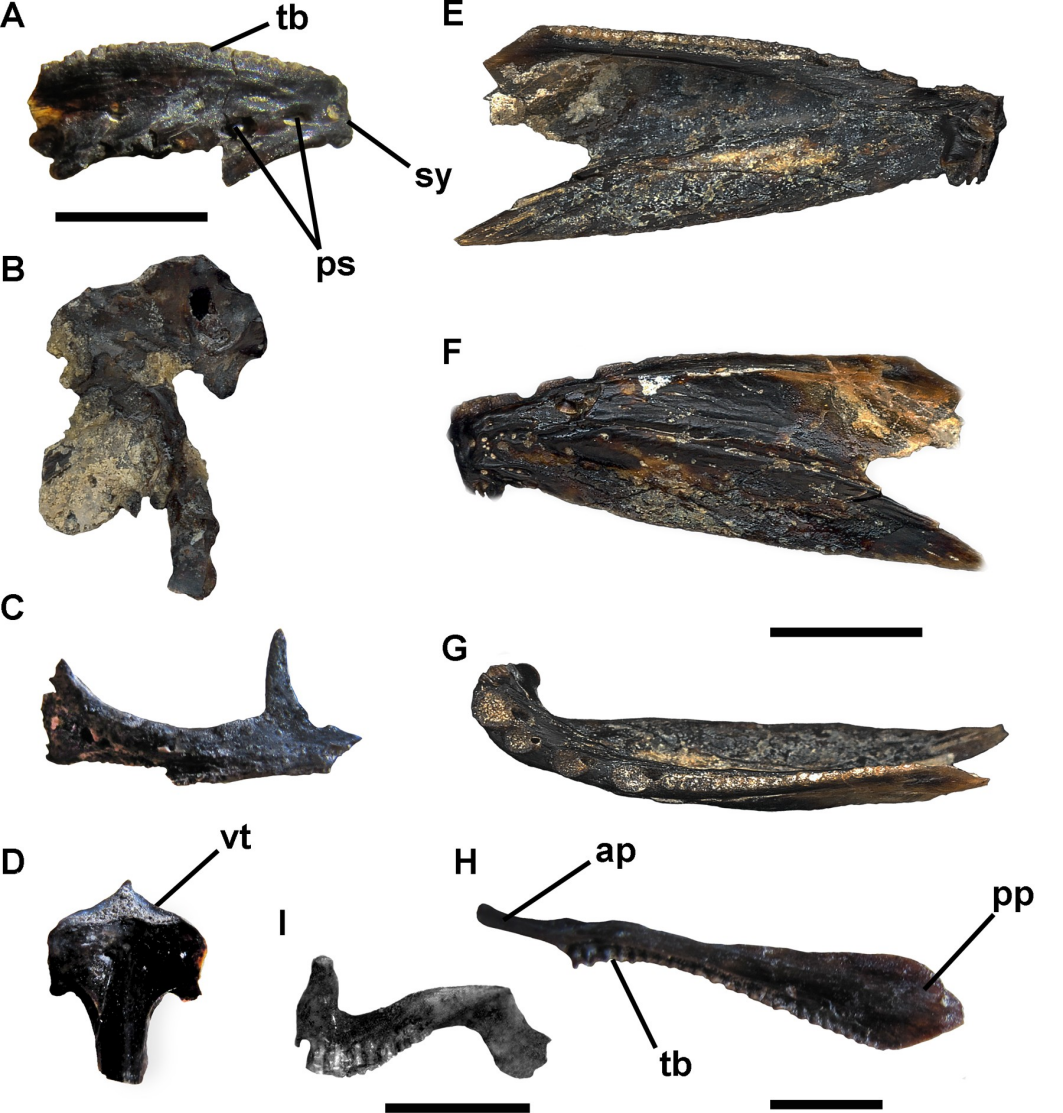
A-D, *Odontesthes* sp. A, right dentary in lateral view (MLP 04-V-2-385); B, fragmentary right hyomandibular in medial view (MLP 04-V-2-222) C, right premaxilla (MLP 04-V-2-452), and D, vomer (MLP 04-V-2-451) in ventral view; E-G, *Oligosarcus* sp., left dentary (MLP 04-V-2-318) in E, medial, F, lateral, and G, occlusal views; H, left maxilla (MLP 04-V-2-419) in lateral view; and I, *Jenynsia* sp., left premaxilla (MLP 04-V-2-225) in lateral view. Scale bar: A-H, 10 mm.; G, 5 mm. **Abbreviations**: ap, anterior process; pp, posterior process; sy, dentary symphysis; ps, openings of the lateral line system; tb, tooth bases; vt, vomerine tooth patch.

OrderAtheriniformes Rosen, 1964
Family Atherinopsidae Fowler, 1903
Subfamily Atherinopsinae Fowler, 1903
Tribe Sorgentinini Pianta de Risso & Risso, 1953
Genus *Odontesthes* Evermann & Kendall, 1906
*Odontesthes* sp.
(Fig 6A–6D)

#### Referred specimens

Frontal (MLP 04-V-2-241 to MLP 04-V-2-242); vomer (MLP 04-V-2-451; Fig 6D); dentary bone (MLP 04-V-2-385; Fig 6A); hyomandibular (MLP 04-V-2-222; MLP 04-V-2-243 to 244; Fig 6B); premaxilla (MLP 04-V-2-223; MLP 04-V-2-452); opercle (MLP 04-V-2-224); interopercle (MLP 04-V-2-453); epihial (MLP 04-V-2-240); cleithrum (MLP 04-V-2-446; MLP 04-V-2-239); precaudal vertebrae (MLP 04-V-2-233 to 238) and caudal vertebrae (MLP 04-V-2-227 to 232).

#### Occurrence

Facies Association 2, Lithofacies G and I (late Bonaerean Stage, late middle Pleistocene; [36]).

#### Description and comparisons

Here we add newly recovered remains to those already found for this taxon, including a partial right dentary bone (MLP 04-V-2-385; Fig 6A). This element shows a row of openings of the sensitive mandibular canal, a relatively shallow and smooth mandibular symphysis that forms a ventral chin at its anterior end. In addition, we recovered an almost complete vomer (MLP 04-V-2-451; Fig 6D) showing a slender and transversely elongate patch for the insertion of small teeth on the anterior portion of the ventral surface. The description of a premaxilla (MLP 04-V-2-452; Fig 6C) is omitted because it does not provide any further relevant information [33].

Vomerine teeth are absent in *O*. *hatcheri*, *O*. *humensis*, *O*. *retropinnis*, *O*. *nigricans*, *O*. *gracilis*, and most species of the subgenus *Cauque* [90], whereas they are arranged in two or three patches in species of the subgenus *Odontesthes* [91]. The morphology of MLP 04-V-2-451 and MLP 04-V-2-385 is very similar to that of modern specimens of *O*. *bonariensis*. Currently, it is the only species of freshwater silversides occurring in the icthyogeographic area Bonaerean Watercourses of Atlantic Drainage, where its distribution is exclusively restricted to large watercourses and basins (Quequén Salado and Quequén Grande Rivers, Sauce Grande Lagoon; [33, 54, 73], missing in brooklets and other small water bodies [73].

In the Quaternary of the Pampas Region this genus was previously reported at this site [33] and in the Quequén Salado [31].

Order Cyprinodontiformes Berg, 1940
Family Anablepidae Bonaparte, 1831
Subfamily Anablepinae Garman, 1895
Genus *Jenynsia* Günther, 1866
*Jenynsia* sp.
(Fig 6I)

#### Referred specimens

Premaxilla (MLP 04-V-2-225; Fig 6I); frontal and opercle (MLP 04-V-2-456).

#### Occurrence

Facies Association 2, Lithofacies I (late Bonaerean Stage, late middle Pleistocene [36].

#### Description and comparisons

The opercle is roughly subtriangular in contour. It is relatively wide and shows a straight anterior margin and a slightly convex dorsal one. In medial view, there are two crests that converge towards the hyomandibular condyle, which is located at the anterodorsal corner.

The frontal bone, in dorsal view, shows a shallow concavity near its anterior margin and a poorly defined orbital margin.

Unfortunately, the absence of derived characters in the fossil material precludes any specific assignment. At present, *Jenynsia lineata* is the only species present in the area [68, 73, 74].

Characiformes *sensu* Fink and Fink, 1996
Family Characidae *sensu* Mirande, 2018
Genus *Oligosarcus* Günther, 1864
*Oligosarcus* sp.
(Fig 6E–6H)

#### Referred specimens

Frontal (MLP 04-V-2-408; MLP 04-V-2-420); basioccipital (MLP 04-V-2-427; MLP 04-V-2-429); mesethmoid (MLP 04-V-2-388 to 390); premaxilla (MLP 04-V-2-413); maxilla (MLP 04-V-2-419; MLP 04-V-2-447; MLP 04-V-2-450; MLP 04-V-2-454; Fig 6H); dentary (MLP 04-V-2-318 to 342; MLP 04-V-2-392; Fig 6E–6G); several conical teeth (no number); angular (MLP 04-V-2-396 to 398; MLP 04-V-2-411; MLP 04-V-2-421 to 423); quadrate (MLP 04-V-2-395; MLP 04-V-2-409); hyomandibular (MLP 04-V-2-391); opercle (MLP 04-V-2-426); preopercular (MLP 04-V-2-406 to 407; MLP 04-V-2-410; MLP 04-V-2-424 to 425; MLP 04-V-2-430); ceratohyal (MLP 04-V-2-387; MLP 04-V-2-402 to 404; MLP 04-V-2-415; MLP 04-V-2-417); cleithrum (MLP 04-V-2-393; MLP 04-V-2-399 to 400; MLP 04-V-2-405; MLP 04-V-2-412; MLP 04-V-2-414; MLP 04-V-2-416; MLP 04-V-2-418; MLP 04-V-2-448) and basipterygium (MLP 04-V-2-401; MLP 04-V-2-449).

#### Occurrence

Facies Association 2, Lithofacies I (late Bonaerean Stage, late Middle Pleistocene; [36].

#### Description and comparisons

Here we add a complete maxilla (MLP 04-V-2-419; Fig 6H) to the abundant skeletal material, including dentary bones and teeth referable to *Oligosarcus* previously described ([34]; Fig 6E–6G),. It shows a general bar-like morphology and bears 24 bases for insertion of teeth. The posterior process is markedly expanded dorsoventrally and the anterior process is flat and somewhat expanded at its end [92].

The gross morphology of available elements is indistinguishable from that of extant species closely related to *Oligosarcus jenynsii* currently inhabiting this area. Although the absence of autapomorphic features precludes species identification, the recovered specimens can be distinguished from other species of the genus [34, 92].

Genus and species indeterminate

#### Referred specimens

Incomplete dentary (MLP 04-V-2-458); ceratohyal (MLP 04-V-2-457).

**Occurrence.** Facies Association 2, Lithofacies I (late Bonaerean Stage, late Middle Pleistocene; [36].

#### Description and comparisons

One incomplete fragment is here referred to a small species of the family Characidae (MLP 04-V-2-458). It is short and robust, has a symphysis with folds and shows a marked ornamentation on the outer lateral surface. A canal accommodating replacement teeth can be observed in occlusal view. In addition, the morphology of a ceratohyal (MLP 04-V-2-457) is different from that corresponding to *Oligosarcus*. Although these remains lack clearly derived features, they are similar to those found in small characids (e.g., genera *Bryconamericus* and *Cheirodon*).

## Discussion

### Taxonomic composition of the Pleistocene palaeoichthyofauna from Centinela del Mar

#### Siluriformes

The extant species that are widely distributed in the icthyogeographic area of Bonaerean Watercourses of the Atlantic Drainage are restricted to the Callichthyidae *Corydoras paleatus* and the Heptapteridae *Pimelodella laticeps* and *Rhamdia quelen* [54, 73, 93]. The material recovered from Centinela del Mar reveals that the diversity of late Middle Pleistocene genera of Siluriformes is nearly indistinguishable to that found in present-day basins of the region. *Rhamdia* is the fish with the largest body size at this site. The most abundant species of the fossil assemblage is referable to *Corydoras*, which is represented by most skeletal elements. These specimens are relatively small, as is usual for *Corydoras*; this finding is in disagreement with the larger specimens of Corydoradinae described for the late Pleistocene locality of Quequén Salado River [29, 31].

#### Atheriniformes

The remains of silversides are relatively abundant in the fossil assemblage and although all the elements are fragmented, their characters allow us to assign them to the genus *Odontesthes* [33]. Currently, it is the only species of freshwater silversides occurring in the icthyogeographic area of Bonaerean Watercourses of the Atlantic Drainage, where its distribution is exclusively restricted to large watercourses and basins (Quequén Salado and Quequén Grande Rivers, Sauce Grande lagoon; [31, 54, 73], missing in brooklets and other small water bodies [73].

In addition to the Centinela del Mar locality, remains of late Pleistocene silversides were also described for the locality of Quequén Salado [31].

#### Cyprinodontiformes

A frontal and an opercle (MLP 04-V-2-456) are added to the single record of a premaxilla referable to the genus *Jenynsia*, which was previously found in Centinela del Mar ([35]; Fig 6I). Unfortunately, the absence of derived characters in the fossil material precludes any specific assignment.

At present, *Jenynsia lineata* is one of the most abundant fishes in the icthyogeographic area of Bonaerean Watercourses of Atlantic Drainage [54, 73].

#### Characiformes

Most of the material collected from Centinela del Mar belongs to an unidentified species of the genus *Oligosarcus* ([34]; Fig 6E–6G). Its remains are very abundant and comprise a large number of anatomical units.

Here we add a dentary fragment and a ceratohyal referable to a small species of the family Characidae (MLP 04-V-2-458 and MLP04-V-2-457). Their morphology differs from that of *Oligosarcus*, and resembles that of small taxa as *Bryconamericus* and *Cheirodon*.

At present, four species of Characidae have been recognised for the icthyogeographic area Bonaerean Watercourses of the Atlantic Drainage: *Oligosarcus jenynsii*, widely distributed in the area, and the “mojarras” *Cheirodon interruptus*, *Astyanax pampa* and *Bryconamericus iheringi* [54, 73, 94].

Based on the considerations stated above, the palaeoichthyofauna from Centinela del Mar emerges as one of the most complete and best known fossil fish assemblages of the Pleistocene of the Pampean Region. This provides the first opportunity to compare extant with Pleistocene fish communities in the area, allowing for palaeoenvironmental and palaeobiogeographic inferences.

### Palaeoenvironmental implications

Since the beginning of the palaeontological investigations carried out in South America, the Pampean Region stood out by yielding abundant remains of megamammals during the Pleistocene (*e*.*g*., [2, 7, 8]), while information on fishes is scarce. Taking into account the remarkable dissimilarities between Pleistocene and extant mammals, it is reasonable to expect that the ichthyofauna contemporaneous with that fauna might be very different from the one found today in the area. However, this assumption is not supported by the fossil record. The continental palaeoichthyofauna from the late Middle Pleistocene of Centinela del Mar (*i*.*e*., *Odontesthes* sp., *Jenynsia* sp., *Corydoras*, *Pimelodella* sp., *Rhamdia* sp., *Oligosarcus* sp. and undetermined Characidae; Table 1) is taxonomically congruent with recent fishes in the study area, with no evidence of important chorological changes. In addition, the fossil record lacks distinctive lineages of taxa occurring presently further north [54, 73]. In this sense, the fossil assemblage from Centinela del Mar resembles those reported from late Pleistocene sites in southern Buenos Aires Province. This is the case for the Quequén Salado River site, which includes mixhoaline species and also specimens of *Rhamdia*, *Synbranchus* and *Corydoras*; the remains belonging to the latter include both the morph described here for Centinela del Mar and a very large morph slightly different from the extant *C*. *paleatus* [29, 31]. Another examples are given by the Paso Otero site in the Quequén Grande River, which includes remains of *Rhamdia* [24, 81] and the La Moderna Ranch site in the Azul stream basin, where *Pimelodella* and *Corydoras* were reported [24, 81]). In contrast, the recovered ichthyofaunas from the Miocene and Pliocene of the southern Pampean region include indeterminate characids [95, 96] and lineages either extinct or currently distributed in distant regions [97, 98, 99], indicating differences with extant fish assemblages. In sum, the composition of the fossil ichthyofaunal assemblage in Centinela del Mar is indistinguishable from that of extant communities in the study area, as previously reported for other fossiliferous Pleistocene sites in the southern Pampean region [100].

**Table 1:** Late middle Pleistocene fish diversity recovered in Centinela del Mar locality.

Order	Taxon	Lithofacies	Environment	References
Perciformes	*Micropogonias furnieri*	K	brackish	Bogan & Cenizo (2008)
Cyprinodontiformes	*Jenynsia* sp.	I	lacustrine	Bogan *et al*.(2008), this work
Siluriformes	*Pimelodella* sp.	I	lacustrine	This work
	*Rhamdia* sp.	I	lacustrine	This work
	*Corydoras* sp.	G—I	fluvio-lacustrine	This work
Characiformes	Characidae indet.	I	lacustrine	This work
	*Oligosarcus* sp.	I	fluvio-lacustrine	Bogan & De los Reyes (2009), this work
Atheriniformes	*Odontesthes* sp.	G—I	fluvio-lacustrine	Bogan *et al*.(2009), this work

The pattern mentioned above is not observed in Cantera Vita site (Bajo San José locality in the Sauce Grande river basin), dated from middle to late Pleistocene in age [24, 28, 80, 100], where there is a unique mixture of Brazilian and Austral lineages. These include the siluriforms *Pimelodella* sp., *Corydoras* cf. *C*. *paleatus* and *Callichthys callichthys* (later assigned to *Callichthys* sp. [100]), which is associated with the percichthyid *Percichthys* sp. Such record constitutes a well-known example of a “non-analog assemblage” [28, 100]. However, a reliable and detailed description of the material is still needed, and the determination of the specimens was not based on discernible anatomical traits [97]. Therefore, the fish assemblage from this fossiliferous site should be taken cautiously until more evidence becomes available.

In addition to the fossil fish material described above, the Lithofacies I of Centinela del Mar has yielded abundant remains of invertebrates, amphibians, reptiles, birds and mammals [36, 37, 38, 101, 102]. From a palaeobiogeographic approach, the presence of anuran remains belonging to the family Pipidae is especially noteworthy because it is currently absent in Argentina [22, 38]. These frogs are strictly aquatic and their dispersal, like for the fishes mentioned above, may exclusively take place through strong floods and across large watercourses.

**Fig 7:**
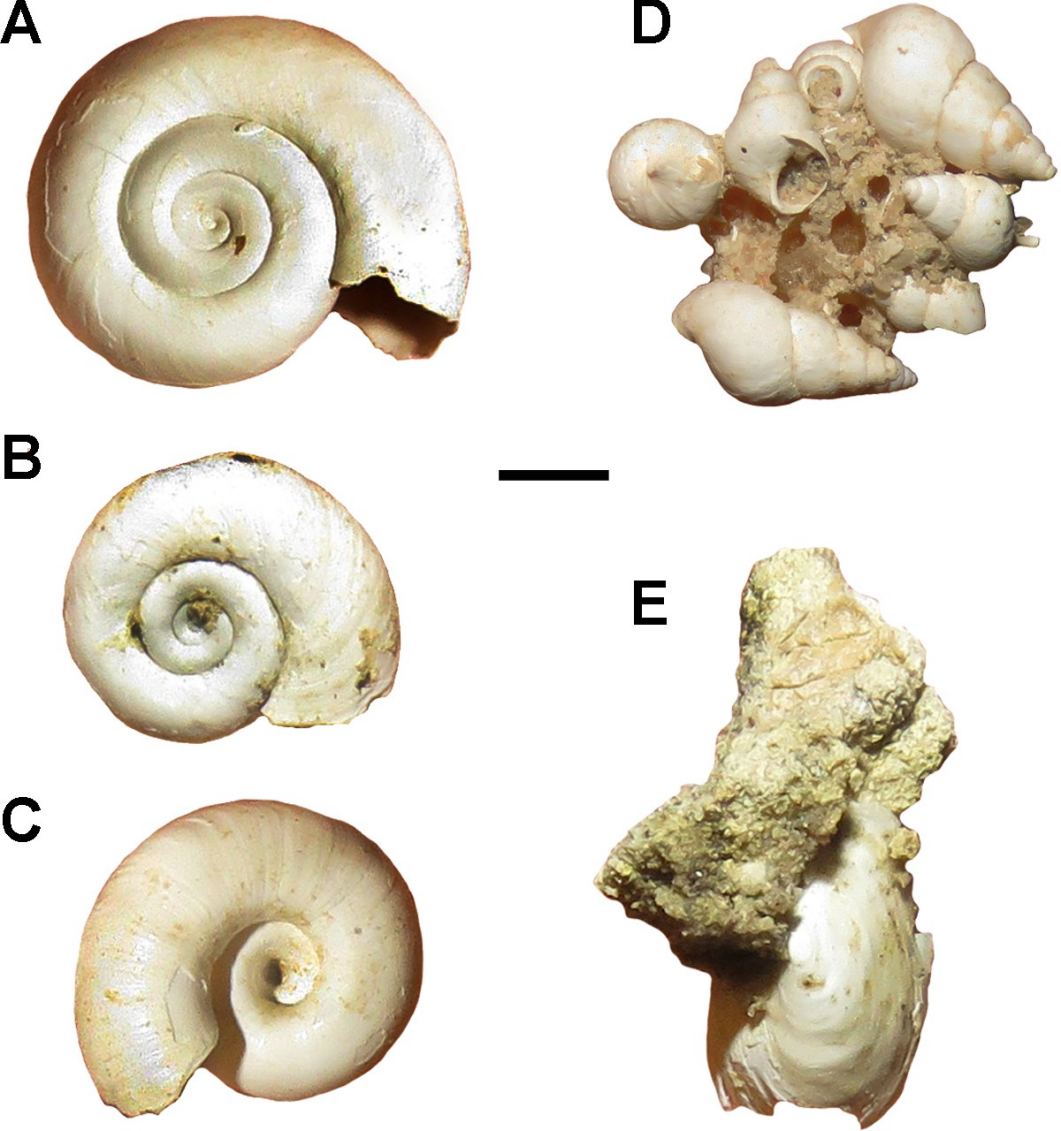
Freshwater molluscs recorded in Centinela del Mar fossiliferous locality at Lithofacies I. A-C, *Biomphalaria* sp. in A,B, dorsal and C, ventral views; D, *Heleobia* sp., several associated shells; E, *Uncancylus* sp., isolated and incomplete shell. Scale bar: 1 mm.

**Fig 8:**
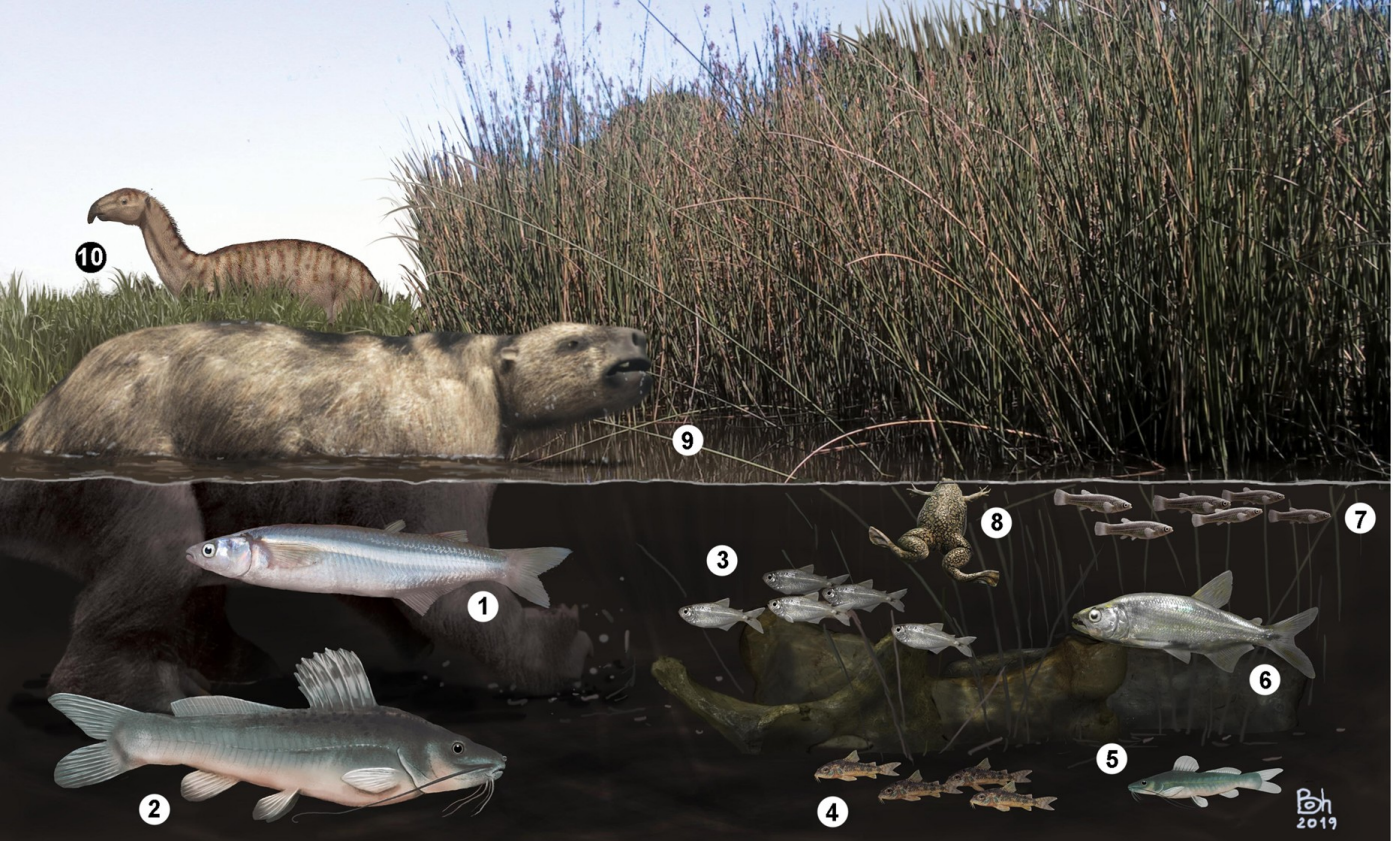
Schematic reconstruction of the lacustrine water body of Lithofacies I in Centinela del Mar fossiliferous locality. 1, *Odontesthes* sp.; 2, *Rhamdia* sp.; 3, Characidae indet.; 4, *Corydoras* sp.; 5, *Pimelodella* sp.; 6, *Oligosarcus* sp.; 7, *Jenynsia* sp.; 8, Pipidae indet.; 9, *Lestodon armatus*; 10, *Macrauchenia patachonica*. Reconstruction by Daniel Boh.

The malacofauna recovered from the Lithofacies I corresponds to three genera of freshwater gastropod taxa (Fig 7) that are common in present-day Pampean lagoons [103, 104, 105]: *Biomphalaria* sp. (Planorbiidae), *Uncancylus* sp. (Ancylidae) and *Heleobia* sp. (Heleobiidae). The fact that species of genera *Uncancylus*, *Biomphalaria* and *Heleobia* are mainly found between leaves of floating plants [103, 104, 105], allows us to infer that the water body had abundant aquatic vegetation (Fig 8). Interestingly, modern representatives of the recovered *Odontesthes* inhabit well-oxygenated open waters of rivers and shallow lakes under conditions of low solar radiation [33], while they are absent in streams and small basins [54]. This is in agreement with the abundant fossil record of ruddy ducks of the genus *Oxyura* [101], whose extant species inhabit water bodies of similar characteristics [106]. As mentioned above, these characteristics coincide with the sedimentological attributes of the host sediments.

In general, the recovered ichthyofaunal specimens are disarticulated and fragmented, well preserved and lack erosive wear marks on the surface caused by transport agents (in contrast with that reported for the late Pleistocene ichthyofauna of Salto and Cantera Los Sauces; [30, 82]). There are some exceptions, such as the finding of two articulated dentaries of *Oligosarcus* sp., incomplete vertebral sequences of fishes, skull elements of *Rhamdia* sp., and skeletal portions of small birds [101]. The predominance of disarticulated elements may be related to the dynamics of the lacustrine system, involving slow water currents leading to low sedimentation rates [107].

Based on the considerations mentioned above and taking as reference the present-day Pampean lagoons, the recovered taxa can be classified into three ecological categories [64].

1- Fishes related to the bottom of a lacustrine water body: i.e., *Corydoras*, *Pimelodella* and juvenile specimens of *Rhamdia* [64]. The former two feed on benthic zooplankton often associated with vegetated bottoms. This feeding category corresponds to juveniles of *Rhamdia* spp., while adults are carnivorous, eating molluscs and other fishes, among others.

2- Open-water (limnetic) fishes, like *Odontesthes* and, to a lesser extent, *Oligosarcus* [64]. Silversides of the genus *Odontesthes* are mainly planktivorous and also feed on crustaceans; specimens older than five years prefer to feed on the gastropod *Heleobia*, while larger ones are piscivorous and may feed on conspecific juveniles.

3- Fishes that frequent vegetated areas, like *Oligosarcus*, *Jenynsia* and small Characidae (*e*.*g*. *Bryconamericus*, *Astyanax* and *Cheirodon*). These taxa predate on small to medium-sized prey and prefer sites with strong water circulation, as does *Corydoras* [64].

### Insights into the evolution of regional basins

Currently, there are a few lacustrine water bodies in the icthyogeographic area of Bonaerean Watercourses of Atlantic Drainage. They are close to the marine littoral and were formed by damming of rivers and streams by coastal dunes. Following an actualistic approach based on sedimentological and palaeontological studies, the deposits of the Lithofacies I (yielding the main fish assemblage analyzed herein) most probably had a similar genesis. This hypothesis is supported by the presence of deposits lying immediately below Lithofacies I (i.e., Lf F-G; Fig 1), which originated by freshwater runoff and mudflows related to fluvial basins [36] and contain fish records that are congruent with these environments, such as Sorgentinini indet. (Atheriniformes) and *Corydoras* (Siluriformes) [108, 33]. In this line of reasoning, Lithofacies I would represent the bottom of a lacustrine water body.

Finally, the unit overlying the Lithofacies I (i.e., Lf K) yielded remains referable to the euryhaline *Micropogonias furnieri* (Sciaenidae, Perciformes; [32]). The Lithofacies K (Pascua Formation *sensu* [43, 36]), likely corresponds to a sandy deposit involving a brackish environment of marine origin.

Most of present-day Bonaerean Watercourses of Atlantic Drainage appear to have been modelled quite recently, driven by the reactivation of former widely-distributed basins probably formed during past periods of heavy rainfall. This is consistent with the record of extensive diamicton deposits that under- and overlie the Lithofacies I of Centinela del Mar (lithofacies F-G and J, [36]) and characterise all the littoral outcrops in the southwest of Buenos Aires Province (*e*.*g*., [109]). Such geological framework may have led to the development of a water body ecologically suitable for the ichthyofauna analysed here.

## Conclusions

Based on sedimentary, taphonomic and palaeofaunal evidence, the Lithofacies I of Centinela del Mar represents a shallow and vegetated lacustrine deposit characterised by relatively clear and well-oxygenated waters and weak water dynamics.

So far, this stratigraphic unit has yielded abundant fossil elements (over 200 bony remains and scales) but a low taxonomic diversity. In fact, only seven freshwater fish taxa have been identified in the assemblage (i.e., *Odontestes* sp., *Jenynsia* sp., *Corydoras*, *Pimelodella* sp., *Rhamdia* sp., *Oligosarcus* sp. and Characidae indet.), the most frequent being *Corydoras* sp. and *Oligosarcus* sp.

The taxonomic composition of the recovered ichthyofauna and malacofauna is similar to that found today in the study area and in lagoons of the Bonaerean Plains between Hills area. This fact, together with the absence of fishes typical of the Paraná-La Plata Rivers Basin (e.g. fishes: Pimelodidae and Anostomidae; molluscs: Ampullariidae and Mycetopodidae), suggest that both basins have been isolated since the Pleistocene.

All the fossils described here belong to the Brazilian lineage and are closely related to extant taxa in the study area. The present-day water bodies included in the Bonaerean Watercourses of Atlantic Drainage also show a low taxonomic diversity. At a regional scale, assemblages composed of more than six taxa have only been recorded in largest aquatic biotopes. Therefore, the number of taxa described in this study suggests that fishes inhabited a relatively large water body, very similar to the typical Pampean lagoons present in the Bonaerean Plains between Hills area.

In addition, the similarity of the fish taxonomic composition of fossil and extant taxa suggests that fish assemblages of the Bonaerean Plains between Hills area have been well-established, at least, since the late Middle Pleistocene. In this regard, the absence of fish species that are typical of water courses closely related to the Paraná-La Plata Rivers Basin is also congruent with the malacological record in the area, suggesting that the freshwater fauna of the Bonaerean Plains between Hills area have been somewhat geographically isolated since the Pleistocene. This may indicate that this area has been well-established since, at least, the middle Pleistocene. Interestingly, haplotype-based analyses in phylogeographic studies of extant fish species (i.e., *Corydoras paleatus* and *Jenynsia lineata*) revealed the presence of lineages that have remained in the area since the late Pleistocene [72, 74]. In the same sense, Frenguelli [48] hypothesised that in the past, the hydrographic pattern in the Bonaerean Plains between Hills area was similar to that of the present time, but with a more extensive development of the valleys due to the effect of heavier rainfalls. This assumption is supported by the geological and palaeoecological information obtained from this study.

The strong parallelism between the fossil fish record from this sector of Buenos Aires Province and the diversity of modern taxa indicates that the ichthyofauna did not suffer significant extinctions during the Pleistocene-Holocene interval, in contrast to what is observed in other vertebrates, especially mammals.

The climatic oscillations occurring during the Pleistocene might have had a great influence on the composition and distribution of modern Pampean vertebrates, mainly mammals. However, our results may indicate that the ichthyofauna in Centinela del Mar has not been significantly affected by these climatic events, supporting the hypothesis, postulated for anurans, that the composition of regional freshwater vertebrates underwent some kind of stasis from the mid-late Pleistocene to the Holocene. This raises important questions about the life history of ectothermic vertebrates in the studied area that need to be tackled in the future.
